# Delirium as a Determinant of Long-Term Cognitive Dysfunction in the Context of Post-intensive Care Syndrome: A Prospective Study in a Latin American Environment

**DOI:** 10.7759/cureus.80578

**Published:** 2025-03-14

**Authors:** Patricia Mesa, Katarzyna Kotfis, Cinthya Lecor, Cecilia Leyes, Angel Banchero, Sylvana De Mattos, Veronica Somma, Maria Orellano, Silvina Favretto, Mariana Barros

**Affiliations:** 1 Intensive Care Unit, Hospital Pasteur, Montevideo, URY; 2 Intensive Care Unit, Hospital Español, Montevideo, URY; 3 Department of Anesthesiology, Intensive Care and Pain Management, Pomeranian Medical University, Szczecin, POL; 4 Psychiatry, Hospital Pasteur, Montevideo, URY; 5 Psychology, Hospital Pasteur, Montevideo, URY; 6 Biostatistics, Faculty of Veterinary Medicine, University of the Republic (Udelar), Montevideo, URY

**Keywords:** cam-icu, cognitive, cognitive impairment, delirium, medical intensive care unit (micu), physical sequelae, post intensive care, psychological

## Abstract

Introduction

Cognitive dysfunction represents a major healthcare concern in the 21st century. Prolonged cognitive dysfunction and concomitant psychological and physical disorders in patients admitted to the intensive care unit (ICU) are components of the post-ICU syndrome (PICS). Notwithstanding the numerous published studies in this area, our work is the first to explore the relationship between PICS and delirium in the ICU in Uruguay. This research underscores the significance and potential of our study, which we believe will make a substantial contribution to this field of research in Latin America.

Objectives

The incidence rates of the cognitive, psychological, and physical sequelae constituting PICS were evaluated, and the relationships between these disorders and delirium in the ICU were studied.

Methods

This was a prospective cohort study in which patients were followed up for one year after admission to the ICU of Hospital Pasteur between 03/01/2017 and 05/31/2017. The pre-ICU condition of each patient was considered in the analysis. An initial telephone interview was conducted using the following scales: the Hamilton scale was used to assess anxiety, the Pfeiffer scale was used to assess cognitive impairment, and the Barthel scale was used to assess activities of daily living (ADLs). In a second face-to-face interview, the Mini-Mental State Examination (MMSE) and the Beck Depression Scale II (BDS-II) were used.

Results

Forty-three patients were divided into two groups: 15 (34%) with delirium in the ICU and 28 (66%) without delirium. The association of delirium with different sequelae was evaluated using the corresponding scales: Pfeiffer scale: Cognitive impairment was observed in 7/13 patients (53%) in the delirium group vs. 0/29 patients (0%) in the non-delirious group (p 0.001); MMSE score: Deterioration was observed in 6/7 patients (86%) in the delirium group vs. 1/7 patients (14%) in the non-delirious group (p 0.007). Cognitive impairment was found in 3/6 patients (50%) who presented with delirium in the ICU, while 1/8 patients (13%) who did not present with delirium experienced cognitive impairment (p = 0.036); Hamilton scale: Anxiety was found in 8/15 patients (57%) in the delirium group and 20/29 patients (68%) in the non-delirious group; BDS-II: Depression was found in 12/12 patients (100%) in the delirium group vs. 27/29 patients (93%) in the non-delirious group (p 0.57). Barthel scale: Dependence on others for ADLs was observed in 3/15 patients (20%) in the delirium group vs. 4/29 patients (14%) in the non-delirious group (p = 0.23).

Conclusions

Cognitive impairment was observed to be associated with delirium in the ICU, opening new avenues for research and possible treatment options. Although dependence on activities of daily living (ADLs) was more common in the delirium group, the difference between the two groups was not significant, highlighting the need for further research to understand the whole picture. Rates of anxiety and depression after ICU stay were also similar between the two groups, providing a baseline for comparison and informing future studies. The study highlights the urgent need for delirium-specific interventions in the ICU to address cognitive dysfunction and improve long-term outcomes in critically ill patients.

## Introduction

Cognitive dysfunction is one of the most important health concerns of the 21st century. Long-term cognitive dysfunction is common in patients admitted to the intensive care unit (ICU), and together with psychological and physical disorders, it is part of post-intensive care unit syndrome (PICS). PICS is an entity that was first described in 2010 by the Critical Care Medicine Society [1]. It encompasses a set of physical, cognitive, and psychological sequelae that patients may experience after discharge from the ICU.

These sequelae lead to impaired quality of life and influence the long-term prognosis of critically ill patients. This syndrome affects many patients discharged from the ICU [1], causing negative impacts on the quality of life for both the survivors and their relatives and generating substantial healthcare costs. The complications associated with PICS can persist for a long time, and patients must be monitored for signs of these sequelae from the time of admission to the ICU. To optimize the care of ICU survivors, it is important to identify during the ICU stay which patients have factors that may increase the risk of developing PICS.

There is a relationship between brain injury and the development of PICS, especially with respect to the cognitive aspects of PICS [1,2]. The most frequently observed forms of acute brain dysfunction in critically ill patients are delirium and coma [2,3]. Delirium is characterized by acute changes in attention and cognition and has a high incidence in ICU patients. Compared to patients who do not experience delirium, patients with delirium in the ICU have higher mortality rates, a longer duration of invasive mechanical ventilation (IMV), longer stays in the ICU, longer hospital stays, and greater long-term cognitive decline [4].

Early interventions can decrease the risk and duration of delirium in the ICU and represent one type of factor that healthcare professionals can influence to prevent long-term cognitive impairment and other aspects of PICS [2,3]. In Uruguay, a prospective cohort study showed that delirium occurred in 80% of patients subjected to IMV [5].

The objectives of the present study were to evaluate the incidence rates of the cognitive, psychological, and physical disorders that comprise PICS and to study the relationship between the development of delirium during the ICU stay and the development of these disorders.

## Materials and methods

Study population and environment 

This descriptive, prospective cohort study followed patients who were admitted to the ICU of Pasteur Hospital between March 1 and May 31, 2017, for one year. The setting was the ICU of Hospital Pasteur in Montevideo, Uruguay. This multipurpose ICU has 25 beds and provides care to both medical and surgical patients.

All surviving patients 12 months after discharge from the ICU who were older than 18 years, who had been admitted to the ICU for at least 24 hours, and who had been assessed with a tool for diagnosing delirium, namely, the confusion assessment method for the intensive care unit (CAM-ICU), were eligible for inclusion, regardless of whether they required IMV [6,7].

Patients with a known and documented diagnosis of neurological pathology were excluded, as were those with severe neuropsychiatric disorders that prevented the communication necessary for the CAM-ICU. The CAM-ICU is less reliable in patients with neurological disorders [8].

All patients who had a sustained Richmond Agitation-Sedation Scale (RASS) score of -4 or -5, that is, those who remained in a coma for their whole stay in the ICU, were excluded because delirium could not be assessed in these patients.

To determine which subjects were alive one year after admission to the ICU, the medical records system of the hospital was consulted, and patients who could not be traced were excluded.

Recorded clinical characteristics

The following characteristics of the patients were recorded during their stay in the ICU: age, sex, personal history, comorbidities, disease severity indicated by the Acute Physiology and Chronic Health Disease Classification System II (APACHE II) score, whether they received IMV, type of medical or surgical pathology, number of days in the ICU, number of days in the hospital, number of days of analgesia and sedation, and diagnosis of delirium (yes/no). The patient's condition before admission to the ICU was recorded.

The population was divided into two groups: those who presented delirium during their stay in the ICU and those who did not. The influence of delirium on the development of cognitive disorders was evaluated separately for the psychological and physical aspects of PICS. This study was carried out by an interdisciplinary team composed of medical psychology graduates and psychiatrists from the Department of Mental Health of Hospital Pasteur, members of the ICU of Hospital Pasteur, nursing assistants and graduates, resident physicians, and coordinators.

Delirium diagnosis 

We used a validated Spanish language version of the CAM-ICU [6,7] as a diagnostic tool. Delirium was diagnosed when the result obtained with the CAM-ICU was positive. 

The presence of delirium was evaluated using the CAM-ICU twice a day during the morning and afternoon shifts by the corresponding nurses and/or resident physicians. Delirium during the ICU stay was defined as at least one positive result on the CAM-ICU [6,7].

Evaluation of the components of PICS

We defined PICS using measures established for the evaluation of cognitive impairment, psychological disorders (depression and anxiety), and physical debilitation, as follows.

Cognitive Impairment

We used the Pfeiffer scale to assess cognitive impairment and the Mini-Mental State Examination (MMSE) to assess cognitive status.

Pfeiffer scale: This scale was developed specifically to detect cognitive impairment in patients older than 18. It consists of 10 questions that assess several basic functions (short- and long-term memory, attention, orientation, knowledge of everyday events, and math ability). The scores were classified as normal (0-2 errors), mild cognitive impairment (3-4 errors), moderate cognitive impairment (5-7 errors), and severe cognitive impairment (8-10 errors) [9].

Mini-mental state examination (MMSE): The MMSE is a practical screening instrument for detecting cognitive alterations, usually administered by a neuropsychologist or psychologist. It is composed of a set of items that measure orientation (personal, spatial, and temporal), short-and long-term memory (fixation and delayed recall), attention, language praxis (verbal and written expression, verbal and written comprehension), and visuo-constructive skills. The scores were classified as follows: normal (greater than 28 points), mild cognitive impairment (between 25 and 27 points), and severe dementia (less than 24 points) [10].

Anxiety

The Hamilton scale was used to assess anxiety [11]. The test consists of a 14-item questionnaire that assesses the symptoms and behavior presented by the patient while taking the test. The 14 items are all scored from 0 to 4. A final score ≥18 indicated a state of anxiety.

Depression

Depression was assessed using the Beck Depression Inventory II (BDI-2). It is administered in person by professional psychologists or psychiatrists. It aims to identify and measure the severity of typical symptoms of depression in adults and adolescents aged ≥13 years. The items on the BDI-2 are consistent with the criteria outlined in the Diagnostic and Statistical Manual of Mental Disorders IV (DSM-IV) for the diagnosis of depressive disorders. The test consists of 21 items with a total score ranging from 0 to 63. The results are classified as mild depression (14-19 points), moderate depression (20-28 points), or severe depression (29-63 points) [12].

Physical Disability

Physical disability was measured using the Barthel scale [13,14], which assesses basic ADLs. The Barthel scale measures a person's ability to perform 10 basic ADLs. This method provides a quantitative estimate of the individual's degree of independence in ADLs. This scale is also known as the Maryland Disability Index.

The patients were classified into five categories: total dependence (0-20 points), severe dependence (21-60 points), moderate dependence (61-90 points), low dependence (91-99 points), and independence (100 points). The patient's condition was rated before admission to the ICU using the same scale. We scored the patients by administering this questionnaire to the patient or the patient's family and/or consulting the patient's medical records.

Interviews

Two types of interviews were carried out: An initial telephone interview and a subsequent face-to-face interview. During the initial telephone interview, we explained the goals of the post-ICU syndrome evaluation to the patients. The patients were invited to participate, and informed consent was obtained. Once the patients completed the telephone interview, they were invited to a second in-person interview.

In the telephone interview, the Pfeiffer scale was administered. To assess cognitive status, the Hamilton scale was administered to assess anxiety, and the Barthel index was used to assess independence in ADLs. In the second face-to-face interview, the other questionnaires were administered. The MMSE was used to assess cognitive impairment, and the BDI-2 was used to assess depression.

Relationships between delirium and the different components of PICS

After the interviews were conducted, the data obtained from the different scales for each patient were correlated with the presence or absence of delirium during the ICU stay.

Ethical considerations

The Research Ethics Committee of Hospital Pasteur, State Administration of Health Services (ASSE), Montevideo, Uruguay, approved the study (Minutes 29/006/3/696/2017/0/0). All enrolled patients signed an informed consent form.

Statistical analysis

Demographic and clinical variables are summarized using descriptive statistics. Continuous variables are described as the mean and standard deviation (SD) (for normally distributed data) or the median and interquartile range (for nonnormally distributed data). The normality of the distribution for each variable was evaluated by the Kolmogorov‒Smirnov test. Continuous variables were compared between the delirium and non-delirium groups by student's t-test for independent samples if the data were normally distributed and the Mann‒Whitney U test otherwise. Qualitative variables were compared using the chi-squared test or Fisher's exact test. P < 0.05 was considered to indicate statistical significance.

A power calculation was incorporated into the study to account for the sample size, as it was hypothesized that a small cohort could affect statistical significance. The statistical power of the analysis was determined to be 0.47, with a significance level (alpha) set at 0.05.

A non-probabilistic convenience sample was obtained. The sample size was determined by the number of patients admitted to the Pasteur ICU.

## Results

**Figure 1:**
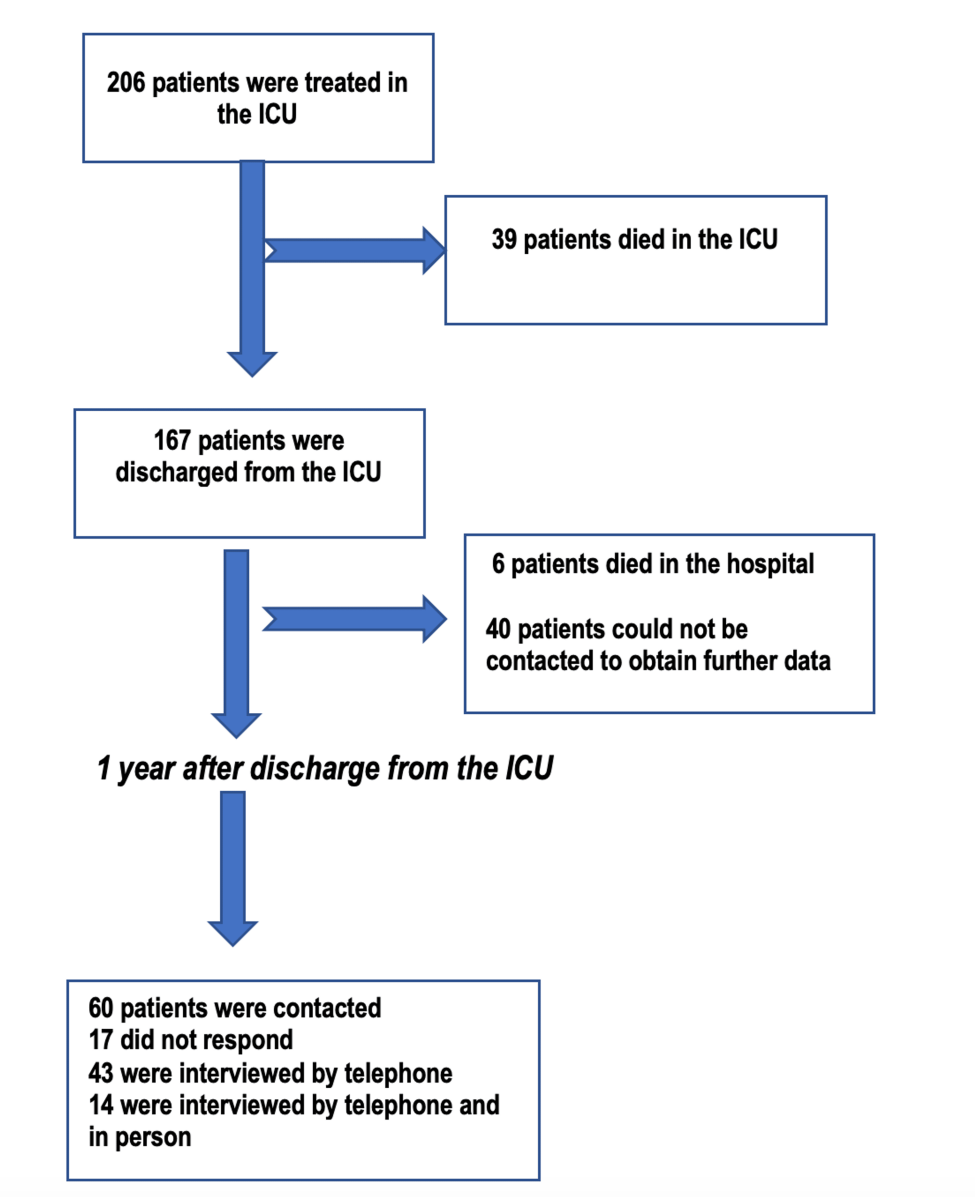
Flowchart of the patient selection process ICU: Intensive care unit

Between March 1 and May 31, 2017, 206 patients were treated in our ICU and were assessed with the CAM-ICU to diagnose delirium. Figure 1 shows the patient selection flowchart. During their stay in the ICU, 39/206 (18%) patients died, and the other 167 were discharged from the ICU, six of whom died in the hospital (Figure 1). Of the 161 patients discharged from the hospital, 43 were interviewed by phone one year after discharge from the ICU. 

Patients we could not contact were not interviewed by telephone or included. All 43 patients who completed the telephone interview were evaluated for PICS with the Pfeiffer, Hamilton, and Barthel scales. These same patients were invited to participate in a second, face-to-face interview in which two other scales were also used: the MMSE to assess cognitive impairment and the BDI-2 to assess depression. Fourteen patients participated in this face-to-face interview.

The 43 patients who completed the telephone interview were divided into two groups according to whether they experienced delirium in the ICU. Of these 43 patients, 15 (34%) presented with delirium in the ICU, and the other 28 (66%) did not. Table 1 shows the general characteristics of the 43 patients who completed the telephone interview and were separated into the delirium and non-delirium groups.

**Table 1:** Baseline characteristics and clinical outcomes according to delirium status during ICU stay of the study population with PICS APACHE II: Acute Physiology and Chronic Health Evaluation; ICU: Intensive Care Unit; PICS: Post-intensive care unit syndrome. Values are expressed as n (%), mean (SD: standard deviation), or median (IQR: interquartile range). * Student's t-test for independent samples; ** Mann–Whitney U test for independent samples. *** Chi-squared test.

Variable	All Patients (n=43)	No Delirium (n=28)	Delirium (n=15)	p-value
Age, mean (SD)	55 (17)	53 (16)	58 (19)	0.30*
Male, n (%)	27 (61)	17 (61)	10 (67)	0.75***
Tobacco Use, n (%)	29 (65)	17 (61)	12 (80)	0.31***
Drug Abuse, n (%)	5 (12)	4 (14)	1 (7)	0.64***
Psychiatric Pathology, n (%)	10 (28)	5 (18)	5 (33)	0.28***
APACHE II Score, mean (SD	15 (6)	15 (6)	24 (11)	0.003*
Mechanical Ventilation, n (%)	21 (49)	8 (29)	13 (87)	<0.001***
Pathology Medical, n (%)	28 (65)	18 (64)	10 (67)	1.00***
Pathology Surgical, n (%)	15 (35)	10 (36)	5 (33)	1.00***
In-ICU Length of Stay (days), mean (SD)	6 (5)	5 (6)	7 (4)	0.42*
In-Hospital Length of Stay (days), mean (SD)	17 (12)	16 (11)	20 (14)	0.33*
Mechanical Ventilation (days), mean (SD)	1 (2)	1 (2)	3 (3)	0.01**
Analgesia (days), mean (SD)	1 (1)	1 (1)	2 (1)	0.011**
Sedation (days), mean (SD)	1 (1)	0 (1)	1 (1)	0.003**

After administering these scales and correlating the results with the occurrence of delirium during the ICU stay, the Pfeiffer and MMSE scores were greater for patients who presented with delirium than for those who did not. When cognitive impairment was evaluated via the Pfeiffer scale, only the group of patients who presented with delirium during their stay in the ICU presented some degree of cognitive impairment.

While in the group of patients who did not present delirium during their ICU stay, none presented cognitive impairment (Table 2). The duration of delirium was five days for patients who developed cognitive impairment and one day for those who did not develop cognitive impairment (p < 0.001).

**Table 2:** Relationship between delirium during the ICU stay and cognitive impairment according to the Pfeiffer scale ICU: intensive care unit; CAM-ICU: Confusion assessment method for the intensive care unit No Delirium: Patients who did not present with delirium during the ICU stay. Delirium: Patients who presented with delirium during their stay in the ICU. Delirium was defined as at least one positive result on the CAM-ICU during the ICU stay. The Pfeiffer scale, which assesses cognitive impairment and consists of 10 questions, is scored as follows: normal (0-2 errors), mild cognitive impairment (3-4 errors), moderate cognitive impairment (5-7 errors), and severe cognitive impairment (8-10 errors). *** The chi-squared test was performed on the data collected during the telephone interviews. Values are expressed as n (%). Note: One patient out of the total 43 could not be assessed. *** The chi-squared test was performed.

Pfeiffer Classification	Total (N = 43)	No Delirium (n = 28)	Delirium (n=13)	p-value
Mild Cognitive Impairment, n (%)	3	0 (0)	3 (23)	<0.001***
Moderate Cognitive Impairment, n (%)	4	0 (0)	4 (30)	Not applicable
Normal, n (%)	35	28 (100)	6 (46)	Not applicable

Among the 14 patients who completed the face-to-face interviews, six presented with delirium, and eight did not. According to the MMSE, 6/7 patients (86%) with delirium presented some degree of deterioration, compared with 1/7 patients (14%) without delirium (p = 0.007).

We next evaluated cognitive impairment and dementia. Cognitive impairment was found in 3/6 patients (50%) who presented with delirium in the ICU, while 1/8 patients (13%) who did not present with delirium experienced cognitive impairment (p = 0.036).

Dementia was found in 2/6 patients (33%) in the delirium group vs. 0/8 patients (0%) in the non-delirium group (p = 0.16) (Table 3). Cognitive impairment was associated with the presence of delirium in the ICU according to both the Pfeiffer scale (p <0.001) and the MMSE (p = 0.007) (see Table 4). The duration of delirium was four days in patients who developed cognitive impairment, compared to 1 day in those without cognitive impairment (p = 0.005).

**Table 3:** Relationship between delirium during ICU stay and cognitive impairment according to the results of the MMSE performed in the face-to-face interview. ICU: intensive care unit; MMSE: Mini-Mental State Examination; CAM-ICU: Confusion assessment method for the intensive care unit Notes: Values are expressed as n: number of patients. Only 14 patients could be assessed with the MMSE by face-to-face interview. No Delirium: Patients who did not present with delirium during the ICU stay. Delirium: Patients who presented with delirium during their stay in the ICU. Delirium was defined as at least one positive result on the CAM-ICU during the ICU stay. MMSE detects cognitive alterations with the following values: <24 indicates dementia; 25-27 indicates mild cognitive impairment; >28 indicates normal (no cognitive impairment). The MMSE was used only in face-to-face interviews. *** The chi-squared test was performed on the data collected in the face-to-face interviews. Values are expressed as n: number of patients.

Mini Mental State Examination	All patients (n=14)	No Delirium (n=8)	Delirium (n=6)	p-value
Normal, n	8	7	1	Not applicable
Mild Cognitive Impairment, n	4	1	3	0.036***
Dementia, n	2	0	2	0.16***

When depression was evaluated with the Beck Depression Inventory II (BDI-II), depression was found in 6/6 patients (100%) who experienced delirium and 8/8 patients (100%) without delirium (p = 0.57). That is, 100% of the interviewees presented some degree of depression (Table 5).

**Table 4:** Relationship between delirium and cognitive impairment according to the Pfeiffer scale and the MMSE. ICU: Intensive care unit; MMSE: Mini-Mental State Examination Notes: Values are expressed as n: number of patients. No Delirium: Patients who did not present with delirium during the ICU stay. Delirium: Patients who presented with delirium during their stay in the ICU were classified as having at least one positive result on the confusion assessment method for the intensive care unit (CAM-ICU) during the stay. The Pfeiffer scale, which assesses cognitive impairment and consists of 10 questions, is scored as follows: normal (0-2 errors), mild cognitive impairment (3-4 errors), moderate cognitive impairment (5-7 errors), and severe cognitive impairment (8-10 errors). *** The chi-squared test was performed on the data collected during telephone interviews. Values are expressed as n (%). Note: One patient out of the total 43 could not be assessed. MMSE detects cognitive alterations with the following values: <24 indicates dementia; 25-27 indicates mild cognitive impairment; >28 indicates normal (no cognitive impairment). The MMSE was used only in face-to-face interviews. Only 14 patients could be assessed with the MMSE by face-to-face interview. *** The chi-squared test was performed on the data collected by telephone and face-to-face interviews.

Pfeiffer scale	All patients (n =43)	No Delirium (n =29)	Delirium (n =13)	p-value
Normal	35	29	6	Not applicable
Deterioration	7	0	7	< 0.001***
Mini-Mental State Examination	All patients (n=14)	No Delirium (n=8)	Delirium (n=6)	Not applicable
All patients	14	8	6	Not applicable
Normal	8	7	0	Not applicable
Deterioration	6	1	6	0.007***

**Table 5:** Relationships between delirium during ICU stay and results obtained with the BDI-II. BDI-II: Beck Depression Inventory II; ICU: Intensive care unit; CAM-ICU: Confusion assessment method for the intensive care unit Notes: Values are expressed as n: number of patients and %. *** Chi-squared test. No Delirium: Patients who did not present with delirium during the ICU stay. Delirium: Patients who presented with delirium during their stay in the ICU. Delirium was defined as at least one positive result on the CAM-ICU during the ICU stay. The Beck Depression Inventory II (BDI-II) assesses depression. This scale was applied in a face-to-face interview. It consists of 21 items, with scores ranging from 0 to 63. The results are classified as mild depression (14 to 19 points), moderate depression (20 to 28 points) or severe depression (29-63 points). This instrument was used only in face-to-face interviews (14 patients).

Beck Depression Inventory (BDI-2)	No Delirium (n=8)	Delirium (n=6)	p-value
Minimal depression, n (%)	3 (43)	5 (72)	0.57***
Mild depression, n (%)	2 (29)	1 (14)	Not applicable
Moderate depression, n (%)	0 (0)	0 (0)	Not applicable
Severe depression, n (%)	2 (29)	1 (14)	Not applicable

The results obtained with the Barthel scale to assess ADLs showed the following: dependence in completing ADLs in 5/14 patients (33%) with delirium vs. 9/28 patients (32%) without delirium, but the differences were not statistically significant (p = 0.23) (Table 6).

**Table 6:** Relationships between delirium during ICU stay and the pre- and post-ICU results obtained with the Barthel scales. ICU: Intensive care unit; CAM-ICU: Confusion assessment method for the intensive care unit Notes: Values are expressed as n: number of patients and %. *** Chi-squared test. No Delirium: Patients who did not present with delirium during the ICU stay. ICU: intensive care unit Delirium: Patients who presented with delirium during their stay in the ICU. Delirium was defined as at least one positive result on the CAM-ICU during the ICU stay. The Barthel scale assesses basic activities of daily living, and patients are classified into 5 categories: total dependence (0-20 points), severe dependence (21-60 points), moderate dependence (61-90 points), low dependence (91-99 points) and independence (100 points). Pre-ICU data were collected before admission to the ICU, and post-ICU data were collected 1 year after discharge from the ICU. This survey was carried out by telephone.

Pre-ICU Barthel classification (n=43)	No Delirium (n=28)	Delirium (n=15)	p-value
Low dependence, n (%)	3 (11)	1 (7)	0.78 ***
Moderate dependence, n (%)	1 (4)	0 (0)	Not applicable
Severe dependence, n(%)	1 (4)	2 (13)	Not applicable
Total dependence, n (%)	0 (0)	0 (0)	Not applicable
Independence, n (%)	23 (82)	12 (80)	Not applicable
Post-ICU Barthel classification (n=43)			Not applicable
Low dependence, n (%)	5 (18)	1 (7)	0.23 ***
Moderate dependence, n (%) 2(7)	2 (7)	2 (13)	Not applicable
Severe dependence, n (%)	2 (7)	0 (0)	Not applicable
Total dependence, n (%)	0 (0)	2 (13)	Not applicable
Independence, n (%)	19 (68)	10 (67)	Not applicable

The results obtained with the Hamilton scale revealed anxiety in 9/28 patients (30%) who reported delirium vs. 19/28 patients (67%) without delirium, but the differences were not statistically significant (p = 1.0) (Table 7). Table 7 also shows the relationships between delirium and all aspects of the post-ICU syndrome: cognitive impairment (MMSE and the Pfeiffer scale), depression (BDI-2), anxiety (Hamilton scale), and ADL dependence (Barthel scale).

**Table 7:** Relationships between delirium and all aspects of post-ICU syndrome: cognitive impairment (MMSE and Pfeiffer scale), depression (BDI-2), anxiety (Hamilton scale) and dependence in activities of daily living (ADLs): (Barthel scale). MMSE: Mini-Mental State Examination; ICU: Intensive care unit; BDI-II: Beck Depression Inventory II; ADLs: Activities of daily living Notes: Values are expressed as n: number of patients and %. *** Chi-squared test. No Delirium: Patients who did not present with delirium during the ICU stay. Delirium: Patients who presented with delirium during their stay in the ICU. Delirium was defined as at least one positive result on the CAM-ICU during the ICU stay. The Barthel scale assesses basic activities of daily living, and patients are classified into 5 categories: total dependence (0-20 points), severe dependence (21-60 points), moderate dependence (61-90 points), low dependence (91-99 points) and independence (100 points). Pre-ICU data were collected before admission to the ICU, and post-ICU data were collected one year after discharge from the ICU. This survey was carried out by telephone. The Pfeiffer scale consists of 10 questions, which are classified according to the following errors: normal (0-2 errors), mild cognitive impairment (3-4 errors), moderate cognitive impairment (5-7 errors), and severe cognitive impairment (8-10 errors). This survey was carried out by telephone. The BDI-II assesses depression. The test consists of 21 items, with scores ranging from 0 to 63. The results are classified as mild depression (14 to 19 points), moderate depression (20 to 28 points), or severe depression (29-63 points). This instrument was used only in face-to-face interviews. MMSE detects cognitive alterations as follows: <24, dementia; 25-27, mild cognitive impairment; and > 28, normal cognition. This instrument was used only in face-to-face interviews. The Hamilton scale is used to evaluate anxiety, and it consists of a questionnaire with 14 items referring to the symptoms.

Scale	Scale outcome	No Delirium	Delirium	p-value
Scale	Outcome	No	Yes	Not applicable
Pfeiffer scale	Normal	28 (100%)	10 (71%)	<0.001***
Pfeiffer scale	Deterioration	0 (0%)	4 (29%)	Not applicable
MMSE	Normal	7 (87%)	1 (17%)	0.007***
MMSE	Deterioration	1 (13%)	5 (83%)	Not applicable
BDI-2	Normal	0 (0%)	0 (0%)	1.0***
BDI-2	Deterioration	8 (100%)	6 (100%)	Not applicable
Hamilton scale	Normal	8 (30%)	3 (25%)	1.0***
Hamilton scale	Deterioration	19 (30%)	9 (75%)	Not applicable
Pre-ICU Barthel scale	Normal	23 (82%)	12 (80%)	1.0***
Pre-ICU Barthel scale	Deterioration	5 (18%)	3 (20%)	Not applicable
Post-ICU Barthel scale	Normal	19 (68%)	10 (67%)	1.0***
Post-ICU Barthel scale	Deterioration	9 (32%)	5 (33%)	Not applicable

## Discussion

No published studies have examined the epidemiology of PICS in relation to delirium in the ICU in Uruguay [15]. In our study, all patients evaluated one year after discharge from the ICU presented at least some symptoms of PICS. Cognitive impairment (according to both the Pfeiffer scale and the MMSE) was associated with delirium when the scales were administered by telephone as well as in person.

Ely W. and Pandharipande (2013) [2,4] published a study of brain function in the ICU and showed that the deterioration of cognition after an ICU stay was associated with delirium for patients in all age ranges, and the severity of the deterioration was comparable to that of patients suffering a moderate traumatic injury and/or mild Alzheimer's disease [2].

One-third of the patients in our population who presented with delirium suffered cognitive impairment one year after an ICU stay, in line with the above study, in which delirium and its duration were both risk factors for worse overall cognitive function at 3 and 12 months post-ICU admission [2].

Brummel and Jackson [16] also reported an association between the duration of delirium and cognitive alterations one year after discharge from the ICU in patients who required mechanical ventilation [1,2]. Our work observed an altered cognitive state in 50% of patients. The predisposing factors for cognitive decline after ICU discharge in ventilated patients include age, previous cognitive status, and delirium.

We found a relationship between delirium in the ICU and long-term cognitive impairment, as demonstrated by both the Pfeiffer scale and the MMSE. With the latter, cognitive impairment was detected in one-third of patients who had delirium.

Other authors, such as Girard, Ditus, and Ely, have also described the relationships between different cognitive aspects of PICS. They argue that the observed cognitive deterioration is due to brain injury, which can vary in severity and usually affects different domains of cognition, with delirium being one of the most important risk factors [17].

In a systematic review of potentially modifiable risk factors for cognitive impairment among critically ill individuals, Sakusic et al. [18] found that 6 out of 9 included studies reported associations of delirium and its duration with long-term cognitive impairment after an ICU stay. They found no relationship between mechanical ventilation, sedation, hypoxia, or other factors with cognitive impairment.

The strong association between delirium in the ICU and long-term cognitive impairment that has been demonstrated in multiple published studies is in line with the results of our work [19-21]. The cognitive impairment observed in this study can be contextualized within the findings of other multicenter trials, including the long-term cognitive impairment after critical illness (P.P. Pandharipande et al. [2]) study. In this study, 34% of patients with delirium exhibited cognitive impairment at 12 months following assessment, which is comparable to mild Alzheimer's disease.

The implementation of delirium prevention strategies may prove effective in reducing the incidence of delirium and associated adverse outcomes, such as cognitive decline. Consequently, delirium may be regarded as a modifiable risk factor for dementia, and interventions that prevent and minimize delirium may also reduce or prevent long-term cognitive impairment [21].

Regarding the psychological aspects of PICS, depression was found in all patients, regardless of whether they presented with delirium. Previous studies have shown that at least one-third of patients in the general ICU population experience mental health problems within one year after discharge [22]. In 2021, Andrews et al. [23] published a study on delirium, depression, and long-term cognition, with assessments performed using the BDI-2, and they reported that a history of depression before admission to the ICU was associated with greater severity of depressive symptoms one year after discharge from the ICU. One weakness of our work is that we did not have data on the history of depression in the patients included in this study [23]. However, Andrews et al. found no association between a personal history of depression and cognitive disorders at 12 months after discharge.

Nordnees et al. [24] reported a relationship between depression and long-term cognitive decline in ICU survivors. All our patients presented with some symptoms of depression upon discharge from the ICU, although there are other factors related to delirium that may influence the development of depression after an ICU stay [24].

Notably, we did not link depression with cognitive impairment since 100% of the patients presented with depression. Another weakness of our study was that a diagnosis of depression was made for only the 14 patients who completed the face-to-face interviews; therefore, the sample size was small. Additional studies on this topic that include more patients are needed.

In a study of 821 patients, Jackson et al. reported the occurrence of depression in 33% of the patients at 12 months post-discharge from the ICU [25]. They also observed that depression was 5 times more common than traumatic panic disorder after critical illness and was driven by somatic symptoms rather than cognitive-affective symptoms, which suggests that it may be related to PICS, among other causes [26].

Anxiety was found in 30% of the patients who presented with delirium and 67% of those who did not. Consistent with other studies, anxiety occurred in a high percentage of patients upon discharge from the ICU. No relationship was found between anxiety and delirium. Previous publications have shown that those who experience delirium in the ICU have a greater risk of developing cognitive impairment and psychiatric problems, such as depression and anxiety, after discharge.

Mikkelsen et al. reported that delirium was associated with cognitive impairment and anxiety in long-term ICU survivors [26]. We found a possible association between delirium in the ICU and long-term depression and anxiety, given that most of the patients who presented with delirium in our study developed long-term cognitive impairment, as mentioned above. It is likely that the cognitive state of these patients prevented them from providing adequate responses to the questions about anxiety and depression.

Regarding physical disability, 33% of both delirium patients and non-delirious patients had some degree of disability related to ADLs 12 months after discharge from the ICU. These findings are consistent with the findings of a study by Jackson et al. [25], who also reported that disability related to physical ADLs was present in 32% of individuals at 12 months after an ICU stay.

Different tools have been proposed to improve PICS, such as the use of delirium prevention bundles through the application of non-pharmacological interventions, which are summarized in the ABCDEF bundle [27,28].

The ABCDEF bundle expresses a research-supported framework designed to guide healthcare providers in implementing organizational adjustments with the objective of enhancing the recovery and outcomes of ICU patients. It encompasses a range of components, including A: Assessment Prevention and Management Pain; B: Both Spontaneous Awakening trials and spontaneous breathing trials; C: Choice of Analgesia and Sedation; D: Delirium Assessment, Prevention, and Management; E: Early Mobility and Exercise; and F: Family Engagement and Empowerment [27,28]. Additionally, another prevention tool is the use of diaries by relatives of patients and/or patients who are able to describe their experience and day-to-day situations while they are in the ICU; the patient and the family can use such diaries to understand what happened to them and can serve as a tool to aid recovery [29]. On the other hand, the occurrence of ICU delirium has implications that extend beyond the patient's outcome; it also affects the family. This condition is associated with a series of psychological issues that fall under the phenomenon known as the family variant of PICS (PICS-F). The prospective study conducted by Kotfis et al. indicates that family members of patients with delirium in the ICU experience a notable increase in symptoms of anxiety, depression, and post-traumatic stress disorder (PTSD) compared to family members of patients who do not experience delirium [30].

Limitations of the study

The present study is limited in the following ways: 1) Sample size: The study included a total of 43 patients, which represents a relatively small sample size. This may limit the generalizability of the findings to a larger or more diverse population. 2) Duration of follow-up: Although patients were followed for one year, this duration may prove insufficient to fully capture the long-term effects of PICS. Furthermore, changes occurring after this period were not evaluated. 3) Single-center study: Given that the study is being conducted at a single hospital center, the results may be influenced by clinical practices and demographic characteristics specific to that institution. This limits the extrapolation of results to other settings or populations where different treatment protocols may be used. 4) Logistic regression tests were performed in this study, and no significant values were obtained; this can be considered a study limitation. This may be due to several factors, such as sample size, variability in the data, or the choice of variables included in the model. It is important to mention that this result does not imply that there are no relationships between the variables but that further research may be necessary to obtain a clearer understanding. Future studies may be proposed to address these limitations.

## Conclusions

Cognitive impairment was associated with the occurrence and duration of delirium in the ICU. Although dependence in performing ADLs was more common in the delirium group, the difference was not significant, nor were the differences in the rates of anxiety and depression after the ICU stay. The most important risk factor that health professionals can influence to prevent cognitive dysfunction after an ICU stay is the occurrence of delirium during the ICU stay. Early identification, monitoring, and the application of multiple measures should be performed in a standardized way to reduce the incidence and shorten the duration of delirium, thereby preventing long-term cognitive impairment in survivors of critical illness.

## Figures and Tables

**Figure 2:**
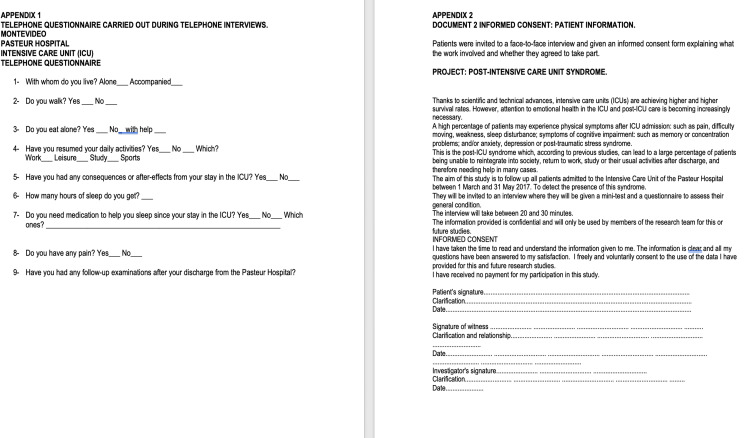
Telephone questionnaire and informed consent

**Figure 3:**
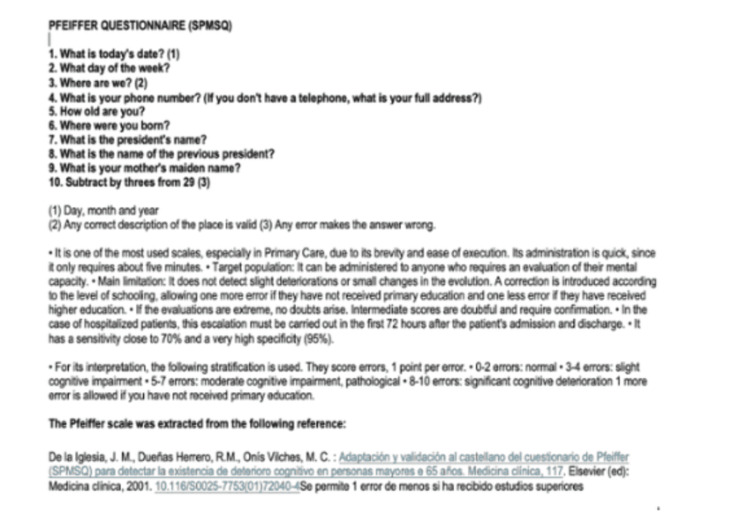
Pfeiffer Questionnaire

**Figure 4:**
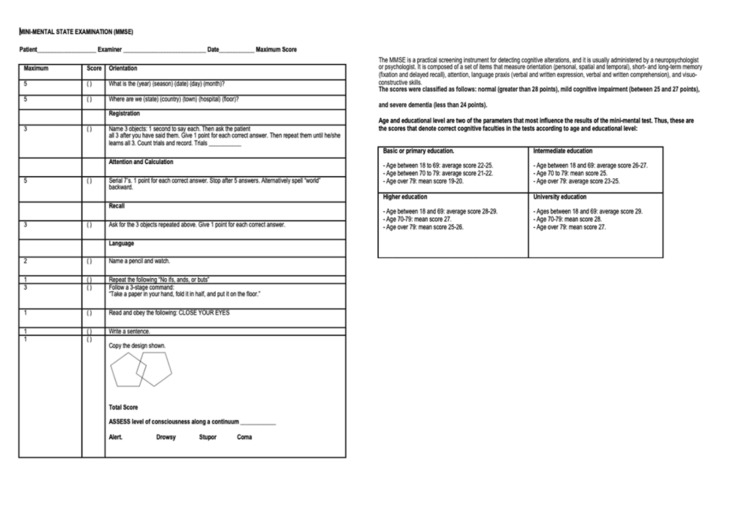
Mini-Mental State Examination (MMSE) [10]

**Figure 5:**
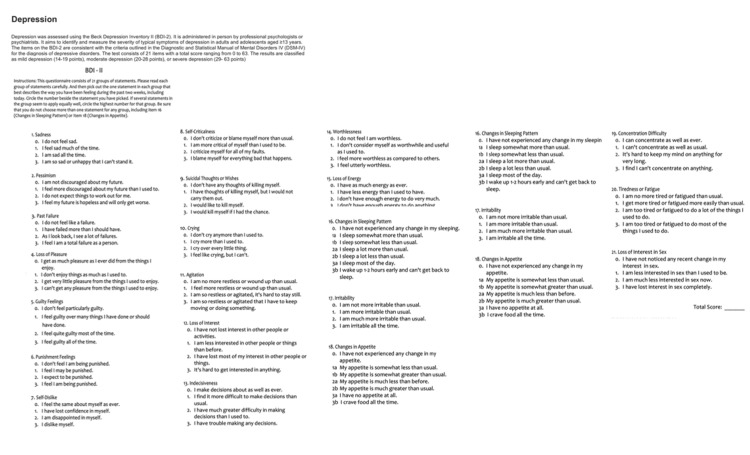
Beck Depression Inventory (BDI-II) [12]

**Figure 6:**
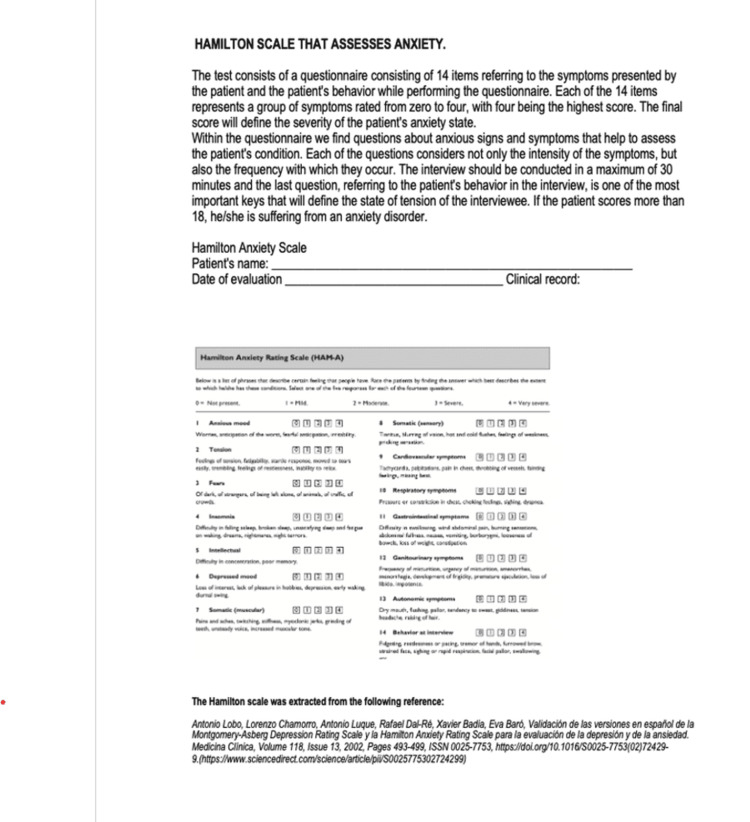
Hamilton Scale [11]

**Figure 7:**
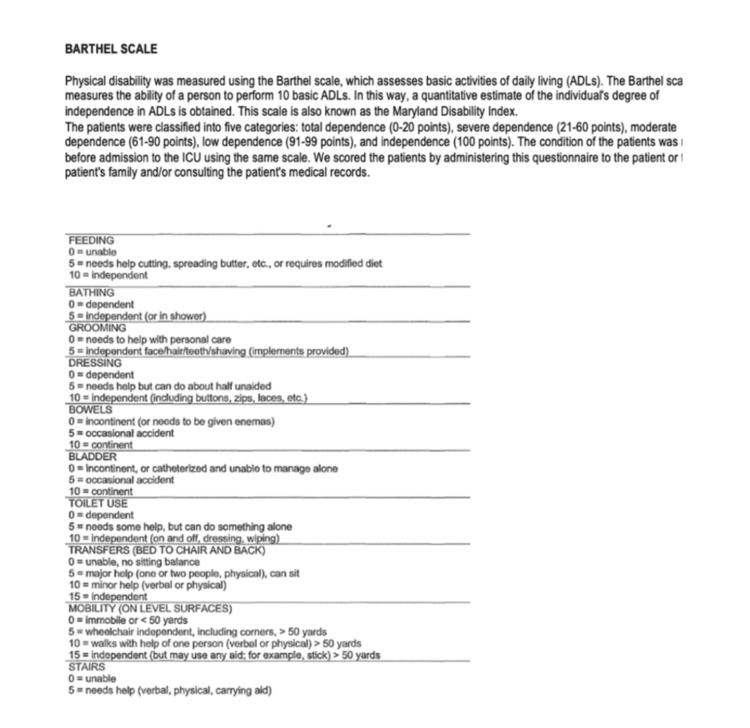
Barthel Scale [14]

## References

[1] Needham DM, Davidson J, Cohen H (2012). Improving long-term outcomes after discharge from intensive care unit: Report from a stakeholders' conference. Crit Care Med.

[2] Pandharipande PP, Girard TD, Jackson JC (2013). Long-term cognitive impairment after critical illness. N Engl J Med.

[3] Miyamoto K, Shibata M, Shima N (2021). Combination of delirium and coma predicts psychiatric symptoms at twelve months in critically ill patients: A longitudinal cohort study. J Crit Care.

[4] Ely EW, Gautam S, Margolin R (2001). The impact of delirium in the intensive care unit on hospital length of stay. Intensive Care Med.

[5] Mesa P, Previgliano IJ, Altez S (2017). Delirium in a Latin American intensive care unit. A prospective cohort study of mechanically ventilated patients. Rev Bras Ter Intensiva.

[6] Ely EW, Margolin R, Francis J (2001). Evaluation of delirium in critically ill patients: Validation of the confusion assessment method for the intensive care unit (CAM-ICU). Crit Care Med.

[7] Tobar E, Romero C, Galleguillos T (2010). Confusion assessment method for diagnosing delirium in ICU patients (CAM-ICU): Cultural adaptation and validation of the Spanish version [Article in Spanish]. Med Intensiva.

[8] van Eijk MM, van den Boogaard M, van Marum RJ (2011). Routine use of the confusion assessment method for the intensive care unit: A multicenter study. Am J Respir Crit Care Med.

[9] De la Iglesia JM, Dueñas Herrero RM, Onís Vilches MC (2001). Cross-cultural adaptation and validation of Pfeiffer’s test (Short Portable Mental Status Questionnaire [SPMSQ]) to screen cognitive impairment in general population aged 65 or older [Article in Spanish]. Med Clin (Barc).

[10] Llamas-Velasco S, Llorente-Ayuso L, Contador I (2015). Spanish versions of the Minimental State Examination (MMSE). Questions for their use in clinical practice [Article in Spanish]. Rev Neurol.

[11] Lobo A, Chamorro L, Luque A (2002). Validation of the Spanish versions of the Montgomery-Asberg depression and Hamilton anxiety rating scales [Article in Spanish]. Med Clin (Barc).

[12] Steer RA, Rissmiller DJ, Beck AT (2000). Use of the Beck Depression Inventory-II with depressed geriatric inpatients. Behav Res Ther.

[13] Sainsbury A, Seebass G, Bansal A, Young JB (2005). Reliability of the Barthel Index when used with older people. Age Ageing.

[14] Collin C, Wade DT, Davies S, Horne V (1988). The Barthel ADL index: A reliability study. Int Disabil Stud.

[15] Mateo Rodríguez E, Puchades Gimeno F, Ezzeddine Angulo A, Asensio Samper J, Saiz Ruiz C, López Alarcón MD (2022). Postintensive care syndrome in COVID-19. Unicentric pilot study [Article in Spanish]. Med Clin (Barc).

[16] Brummel NE, Jackson JC, Pandharipande PP (2014). Delirium in the ICU and subsequent long-term disability among survivors of mechanical ventilation. Crit Care Med.

[17] Girard TD, Dittus RS, Ely EW (2016). Critical illness brain injury. Annu Rev Med.

[18] Sakusic A, O'Horo JC, Dziadzko M (2018). Potentially modifiable risk factors for long-term cognitive impairment after critical illness: A systematic review. Mayo Clin Proc.

[19] Stollings JL, Kotfis K, Chanques G, Pun BT, Pandharipande PP, Ely EW (2021). Delirium in critical illness: Clinical manifestations, outcomes, and management. Intensive Care Med.

[20] Mart MF, Williams Roberson S, Salas B, Pandharipande PP, Ely EW (2021). Prevention and management of delirium in the intensive care unit. Semin Respir Crit Care Med.

[21] Fong TG, Inouye SK (2022). The inter-relationship between delirium and dementia: The importance of delirium prevention. Nat Rev Neurol.

[22] Unoki T, Sakuramoto H, Uemura S (2021). Prevalence of and risk factors for post-intensive care syndrome: Multicenter study of patients living at home after treatment in 12 Japanese intensive care units, SMAP-HoPe study. PLoS One.

[23] Andrews PS, Thompson J, Raman R (2023). Delirium, depression, and long-term cognition. Int Psychogeriatr.

[24] Nordness MF, Bipin Patel M, Erickson CR (2021). Depression predicts long-term cognitive impairment in survivors of critical illness. J Trauma Acute Care Surg.

[25] Jackson JC, Pandharipande PP, Girard TD (2014). Depression, post-traumatic stress disorder, and functional disability in survivors of critical illness in the BRAIN-ICU study: A longitudinal cohort study. Lancet Respir Med.

[26] Mikkelsen ME, Shull WH, Biester RC (2009). Cognitive, mood and quality of life impairments in a select population of ARDS survivors. Respirology.

[27] Devlin JW, Skrobik Y, Gélinas C (2018). Clinical practice guidelines for the prevention and management of pain, agitation/sedation, delirium, immobility, and sleep disruption in adult patients in the ICU. Crit Care Med.

[28] Marra A, Ely EW, Pandharipande PP, Patel MB (2017). The ABCDEF bundle in critical care. Crit Care Clin.

[29] Nydahl P, Deffner T (2021). Use of diaries in intensive care unit delirium patients: German nursing perspectives. Crit Care Nurs Clin North Am.

[30] Kotfis K, Maj P, Szylińska A, Pankowiak M, Reszka E, Ely EW, Marra A (2024). The spectrum of psychological disorders in family members of patients suffering from delirium associated with critical illness: A prospective, observational study. Sci Rep.

